# Comparison of corneal pachymetry evolution after accelerated corneal crosslinking in keratoconus eyes using anterior segment optical coherence tomography and Scheimpflug imaging

**DOI:** 10.1007/s10792-025-03763-4

**Published:** 2025-12-05

**Authors:** Antonia Lang, Berthold Seitz, Cristian Munteanu, Elias Flockerzi

**Affiliations:** Department of Ophthalmology, Saarland University Medical Center, Kirrberger Straße, Building 22, 66424 Homburg, Germany

**Keywords:** Keratoconus, Corneal crosslinking, Corneal thickness, Scheimpflug, Optical coherence tomography

## Abstract

**Purpose:**

This retrospective clinical study compared longitudinal corneal pachymetry measurement evolution after accelerated corneal crosslinking (A-CXL, 9 mW/cm^2^, 10 min) in keratoconus (KC) eyes as measured by Pentacam HR Scheimpflug imaging (Oculus, Germany) and Casia 2 (Tomey, Japan) anterior segment optical coherence tomography (AS-OCT).

**Methods:**

218 eyes covering the full topographic KC (TKC) severity spectrum that underwent A-CXL between 2017 and 2021 were examined at five time points (before, 6 weeks, 6 months, 1 year after A-CXL and at the latest follow-up) using both Pentacam HR and Casia 2. Central (CCT) and thinnest (TCT) corneal thickness were assessed. Corneal densitometry and TKC classification were assessed by Pentacam HR only. Intraoperative CCT evolution was monitored using ultrasound (Pachymeter SP-3000, Tomey, Japan) to ensure that CCT did not fall below 400 µm.

**Results:**

CCT and TCT measured by Pentacam HR were significantly lower than preoperatively during the first postoperative year, while Casia 2 CCT and TCT measurements were not significantly lower than preoperatively at any time. The highest agreement of Pentacam HR and Casia 2 measurements occurred six weeks after A-CXL. Corneal densitometry peaked six weeks after A-CXL and decreased thereafter without reaching pre-A-CXL values. Ultrasound revealed significant CCT thinning during A-CXL.

**Conclusion:**

Corneal Scheimpflug and AS-OCT based thickness measurements are not interchangeable. Corneal thinning after A-CXL detected by Pentacam HR was not observed in Casia 2 measurements. Since higher densitometry values did not correlate with a greater inter-device measurement difference, Scheimpflug-based corneal thickness measurements should be interpreted cautiously during the first year after A-CXL.

## Introduction

Corneal crosslinking (CXL) has become one of the main treatments for Keratoconus (KC), the most common ectatic corneal disorder [1]. KC is characterized by a progressive bilateral asymmetric thinning and steepening of the cornea, especially in its infero-temporal region resulting in the conical shape that gave the disease its name [2, 3]. One recent review estimated the global prevalence to be about 1.38/1000 [4].

Since the first CXL protocol, the standard Dresden protocol (3mW/cm^2^, 30 min), was developed and first introduced in 1998, several different CXL protocol modifications have been developed and introduced into clinical practice [5]. One of the most common variations is the accelerated CXL (A-CXL) (9mW/cm^2^, 10 min), which has been shown to be as effective as standard-CXL in terms of topographic and visual outcomes and can also be performed in children’s eyes [6, 7].

Both, Scheimpflug-based imaging devices such as the Pentacam HR (Pentacam HR®; Oculus, Wetzlar, Germany) and anterior segment optical coherence tomography (AS-OCT) devices such as the Casia 2 (CASIA 2, Tomey, Nagoya, Japan) are commonly used to detect KC, monitor its progression and evaluate corneal pachymetry [8].

Pentacam HR software provides a built-in classification system for KC, the topographic keratoconus classification (TKC), which was adjusted to match the Amsler-Krumeich (AK) classification [9, 10]. TKC considers the index of surface variance (ISV), keratoconus index (KI) and minimum radius (Rmin) and is a staging based on topographic values rather than clinical evaluation [9]. In 2016, Belin and Duncan have published their ABCD staging system for KC corneas, which allows a more precise tomographic KC staging [11, 12], however the TKC system allows a more concise comparison between different KC severity stages at first glance.

Because CXL is a widely accepted KC treatment, its induced postoperative changes in the corneal stroma are an important subject of investigation. One common side effect is the transient formation of haze. Corneal densitometry (CD), measured by the Pentacam HR, is used to quantify the amount of light scattering in the corneal stroma and detect a reduction of corneal transparency [13]. A-CXL has a lower post-surgical haze rate (46.9%) compared to the standard CXL (70.5%) [14] and due to shorter treatment time and its favorable safety profile, it is frequently used in clinical practice.

Several previous studies have evaluated the evolution of thinnest corneal thickness after CXL and have shown a significant postsurgical decrease in thinnest corneal thickness using different Scheimpflug imaging devices [7, 15]. This observed reduction in TCT measurements is clinically relevant, as it may influence further clinical evaluation of treatment success and a possible progression of KC.

Despite widespread use, Scheimpflug imaging devices have shown a lower reliability, particularly in KC corneas [16]. A previous study [17] found significantly higher pachymetric values with Scheimpflug-based measurements compared to AS-OCT in KC patients. Lower thickness measurement with AS-OCT compared to Scheimpflug-based imaging and ultrasound (US) have been reported in other studies as well [18, 19].

These discrepancies might be attributed to the completely different measurement methods used in the devices. The Pentacam HR calculates pachymetry values based on Scheimpflug images of the cornea captured during the examination [20] whereas the AS-OCT measurement is based on interferometry and light reflection [21].

The purpose of this study was to analyze the longitudinal corneal thickness evolution using both Scheimpflug-based measurements and AS-OCT after A-CXL and to evaluate whether the observed corneal thinning detected by Scheimpflug-based devices can be confirmed by AS-OCT measurements. The possible influence of corneal transparency, quantified by CD, on CCT measurements was also analyzed.

## Methods

### Patients and design

This retrospective, monocentric study included 218 patients who underwent A-CXL (9 mW/cm^2^, 10 min) at the department of ophthalmology at the Saarland University Medical Center in Homburg, Germany between 2017 and 2021 [22]. They consisted of 37 female and 181 male KC patients aged between 11 and 64 years (29 ± 11 years, mean ± standard deviation) from the Homburg Keratoconus Center (HKC). 119 right eyes and 99 left eyes were included, only one eye per patient was considered. If both eyes had undergone A-CXL because of KC and good quality measurements (only a quality score “OK” was accepted) were available for both eyes, one eye was randomly selected. A non-contact-lens-wearing period of at least three days prior to the measurements was mandatory and A-CXL was performed in progressive KC corneas defined by a Kmax-increase of more than one diopter per year. Patients that underwent a second intervention such as intracorneal ring segment implantation (ICRS) or penetrating keratoplasty (PKP) after A-CXL were only included in this study before undergoing any second intervention. Eyes with a scarred cornea due to injuries or previous surgery were excluded. This retrospective study is part of the retrospective observational Homburg Keratoconus Center (HKC) study (trial number NCT03923101, U. S. National Institutes of Health, ClinicalTrials.gov), which was approved by the local ethics committee (Ethikkommission bei der Ärztekammer des Saarlandes, reference number 121/20) respecting the principles of the Declaration of Helsinki. Written and informed consent for data analysis was obtained from all patients.

A-CXL was performed after applying topical anesthesia (Oxybuprocain eye drops, Conjucain EDO, Bausch & Lomb, NY, USA) at least three times before abrading the corneal epithelium mechanically using a Hockey knife. Then, a 0.1% riboflavin hydroxypropyl-methylcellulose (HPMC) solution (VibeX Rapid 0.1% solution, Avedro, Waltham, MA, USA) was applied every two minutes for 20 min, thus representing an extended soakage duration. Ultrasonic pachymetry (Pachymeter SP-3000, Tomey, Nagoya, Japan) was performed to ensure that corneal thickness did not fall below 400 μm before UVA irradiation was applied with an irradiance of 9 mW/cm^2^ and a fluence of 5.4 J/cm^2^ for 10 min using the Avedro kxl crosslinking system (Avedro, Waltham, MA, USA). The application of riboflavin solution every two minutes was continued during the irradiation process. During the A-CXL procedure, central corneal thickness was measured using ultrasound (Pachymeter SP-3000) in 114 patients before epithelial abrasion, after epithelial abrasion, after riboflavin application, and after UVA irradiation.

Thinnest corneal thickness (TCT) and central corneal thickness (CCT) were measured preoperatively (n = 147) and six weeks (n = 127), six months (n = 127), 12 months (n = 97) and at their latest follow-up (n = 114), defined as their last examination until July 2023, after A-CXL with Pentacam HR and Casia 2. The pre-A-CXL measurements were taken 1.4 ± 1.3 months (mean ± SD) prior to A-CXL. Measurements took place 1.6 ± 0.4 months (range 0.9 to 3.2 months) after A-CXL for the six weeks values and 3.1 ± 1.4 months (range 3.3 to 9.0 months) after A-CXL for the six months values. The one-year measurements were taken 11.9 ± 1.2 months (range 9.3 to 13.8 months) after A-CXL and the latest follow-up measurements were taken 28.7 ± 15.6 months (range 13.9 to 66.8 months) after A-CXL. Measurements were taken between 9:00 AM and 3:00 PM. This approach was intended to minimize the potential impact of daily fluctuations in corneal thickness.

Because not all patients attended each follow-up visit and only visits with both Pentacam HR and Casia 2 AS-OCT measurements were included, there is a discrepancy between the 218 patients included in the study and the number of values obtained at each time point.

Pentacam HR uses a rotating digital charge coupled device (CCD) camera and a blue light emitting diode (LED) with a wavelength of 475 nm, taking multiple slit illumination images in order to calculate a model of the anterior segment of the eye including a topometric map of the cornea.

Tomey’s Casia 2 AS-OCT uses a swept source laser with a wavelength of 1310 nm to obtain information about the structures and depth of the anterior segment of the eye by detecting and analyzing the intensity and phase of the backscattered light.

Densitometry of the central cornea was measured with Pentacam HR at the same time points as TCT and CCT. The cornea is divided into a central circular zone and three surrounding, concentric annular zones by Pentacam HR, with the first zone covering the central 0–2 mm, the second extending from 2 to 6 mm, the third zone from 6 to 10 mm followed by a limbal zone from 10 to 12 mm. In addition, Pentacam HR divides the cornea into three layers: anterior 120 μm, a central layer of varying thickness, and the posterior 60 μm of the cornea. In this study densitometry data were evaluated from all corneal layers, but only from the central 0–2 mm zone. The densitometry values are expressed in grayscale units (GSU), ranging from 0 (indicating minimal light scattering and maximum transparency) to 100 (indicating minimal transparency and complete scattering).

### Statistical methods

Patient data were collected and managed using a Microsoft Excel Workbook (Microsoft Office 2016, Microsoft Corporation, Redmond, WA, USA). For statistical analysis, SPSS software (version 29.0.1, SPSS/IBM, Inc., Chicago, IL, USA) was used. Unless otherwise stated, data in the descriptive tables and text are presented as the mean value ± standard deviation (SD).

Results with a *p*-value ≤ 0.05 (≤ 5%) were considered statistically significant.

Differences in measurements between AS-OCT and Pentacam HR were analyzed using a paired samples t-test in SPSS software. Results are presented in Bland–Altman plots with a mean value ± 1.96 SD generated using R (version 4.1.3, The R Foundation for Statistical Computing, Vienna, Austria). Tables were generated using Microsoft Excel.

SPSS software was used to analyze the evolution of corneal thickness over time. A general linear model (GLM) was used and a test on least significant difference (LSD) was applied.

The graphs and tables for the stage-dependent analysis of TCT and CCT measurement deviations of Pentacam HR and Casia 2 were generated using SPSS software. An Analysis of Variances ANOVA was used to analyze the data and a post hoc test with Bonferroni adjustment for multiple comparisons was applied.

Intraoperative pachymetry measurements by ultrasound Pachymeter SP-3000 and corneal densitometry values were analyzed using an ANOVA with Bonferroni adjustment for multiple comparisons.

All figures, except for Bland–Altman plots, were generated using SPSS software.

## Results

First, TCT and CCT were analyzed separately for Pentacam HR and Casia 2 AS-OCT over time. The absolute values for each examination and the absolute difference in corneal thickness between the postoperative follow-up examination and the preoperative thickness value are shown in Table 1.

**Table 1:** Mean thinnest corneal thickness (TCT) and central corneal thickness (CCT) ± SD with numbers of eyes for both devices (Pentacam HR and Casia 2), different time points (before A-CXL (PreOP) and at six weeks (6W), six months (6M), one year (1Y) and latest follow-up (Latest) after CXL) and mean delta and relative reduction between PreOP measurements and measurements at the respective time points with p-values according to ANOVA with least significant difference (LSD) test

Date category	Device	TCT ± SD (μm)	Δ PreOP – date category (μm) [relative thinning rate]	n (TCT)	CCT ± SD (μm)	Δ PreOP – date category (μm) [relative thinning rate]	n (CCT)
PreOP	Pentacam HR	460.8 ± 38.0		147	483.8 ± 37.0		147
	Casia 2	444.4 ± 39.6		147	472.0 ± 39.2		147
6W	Pentacam HR	450.1 ± 40.8	10.7* *p* = 0.026 [−2.3%]	127	471.2 ± 37.0	12.6* *p* = 0.006 [−2.6%]	127
	Casia 2	439.3 ± 40.7	5.1 *p* = 0.30 [−1.1%]	127	468.5 ± 38.9	3.5 *p* = 0.48 [−0.7%]	127
6 M	Pentacam HR	448.0 ± 42.6	12.8* *p* = 0.008 [−2.7%]	126	472.4 ± 40.8	11.7* *p* = 0.01 [−2.4%]	126
	Casia 2	437.5 ± 44.0	7.2 *p* = 0.14 [−1.6%]	126	466.5 ± 42.7	4.8 *p* = 0.32 [−1.0%]	126
1Y	Pentacam HR	450.2 ± 37.5	10.8* *p* = 0.038 [−2.3%]	97	473.7 ± 33.4	10.1* *p* = 0.039 [−2.1%]	97
	Casia 2	436.5 ± 37.0	7.9 *p* = 0.14 [−1.8%]	97	466.7 ± 39.3	5.3 *p* = 0.32 [−1.1%]	97
Latest	Pentacam HR	451.2 ± 38.7	9.6 *p* = 0.051 [−2.1%]	114	474.8 ± 37.6	9.1 *p* = 0.053 [−1.9%]	114
	Casia 2	438.1 ± 39.8	6.3 *p* = 0.21 [−1.4%]	114	468.0 ± 40.5	3.9 *p* = 0.44 [−0.8%]	114

* indicating significance on a 0.05 level

**Fig. 1:**
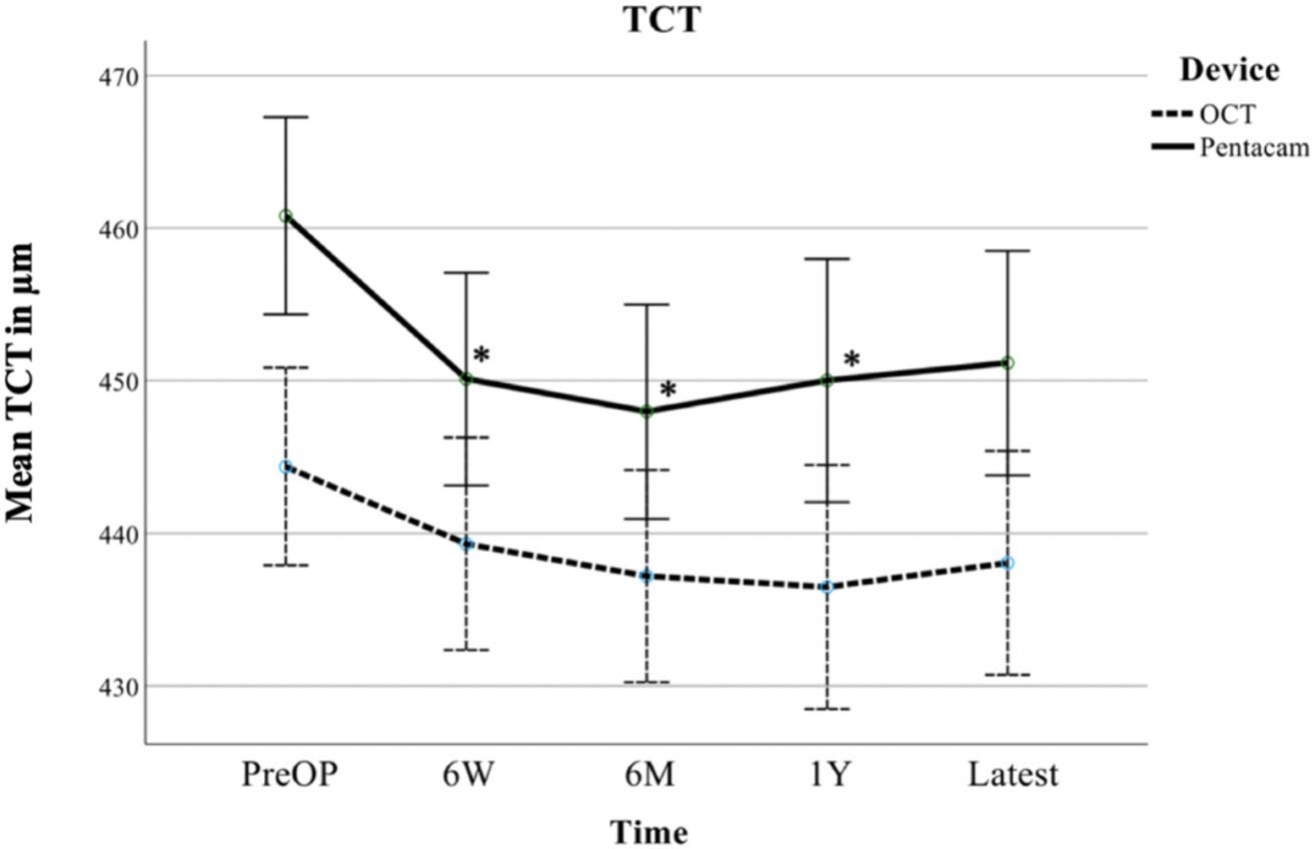
Longitudinal evolution of mean thinnest corneal thickness (TCT) as measured by Pentacam HR (Pentacam) and Casia 2 (OCT), including 95% confidence interval (CI) error bars, given in μm preoperatively (PreOP), at 6 weeks (6W), 6 months (6M), 1 year (1Y) and at the latest follow-up examination (Latest) after A-CXL, * indicating a significant change compared to PreOP measurements on a 0.05 level in ANOVA with LSD test

**Fig. 2:**
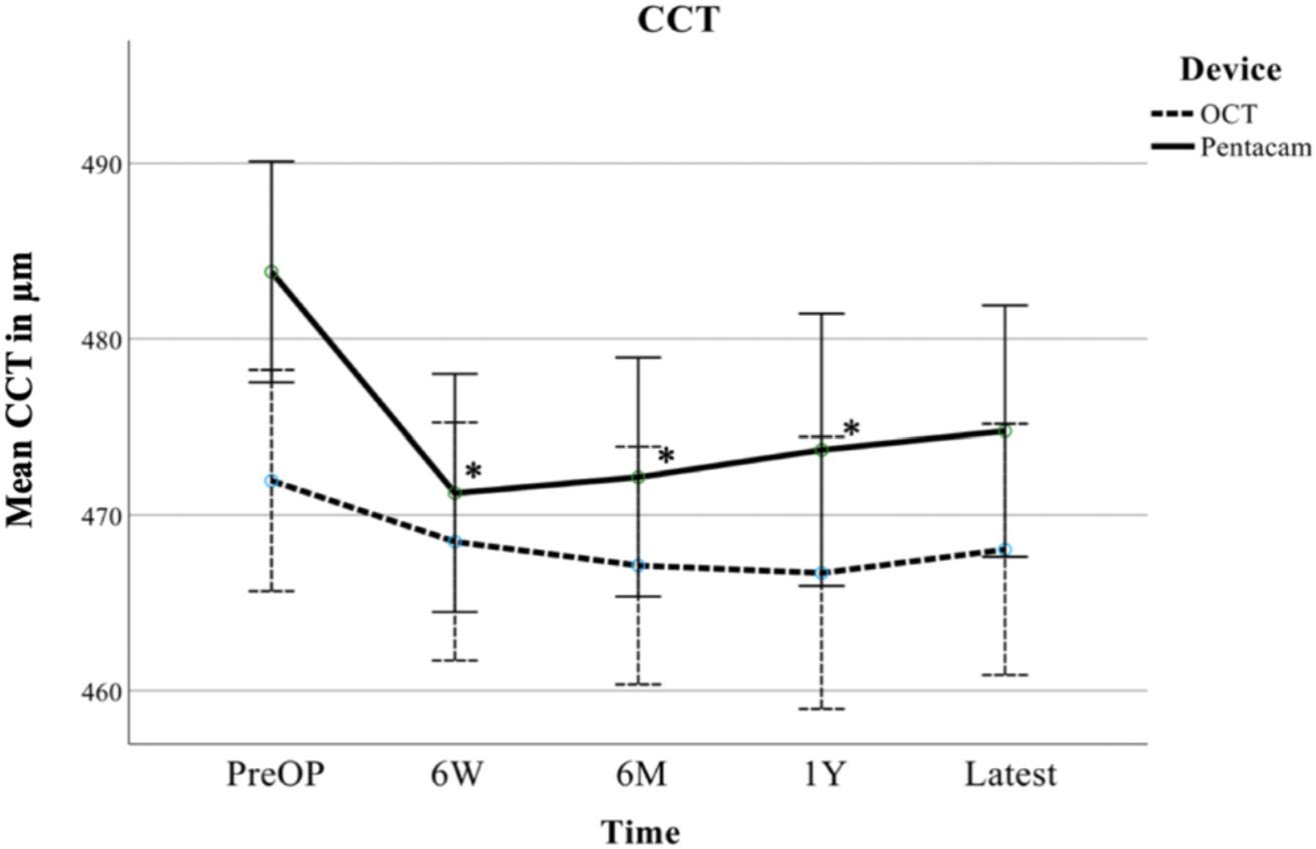
Longitudinal evolution of mean central corneal thickness (CCT) as measured by Pentacam HR (Pentacam) and Casia 2 (OCT), including 95% confidence interval (CI) error bars, given in μm preoperatively (PreOP), at six weeks (6W), six months (6M), one year (1Y) and at the latest follow-up examination (Latest) after A-CXL, * indicating a significant change compared to PreOP measurements on a 0.05 level in ANOVA with LSD test

Absolute measurement values by Pentacam HR and Casia 2 AS-OCT were analyzed over time and are visualized in Fig. 1 for TCT and Fig. 2 for CCT.

The longitudinal analysis of TCT measurements by Pentacam HR showed a significant thinning of the cornea after A-CXL. A mean thinning of 10.7 μm (*p* = 0.026, LSD) was observed after six weeks compared to preoperative values. Thereafter TCT did not change significantly over time, but remained significantly lower than the preoperative values with a mean thinning of 12.8 μm (*p* = 0.008, six months) and 10.8 μm (*p* = 0.038, one year). At the latest follow-up, the thinning of 9.6 μm was no longer significant (*p* = 0.051).

TCT measurements by Casia 2 (AS-OCT) showed lower values at each postoperative follow-up examination, but did not reach statistical significance. Compared to the preoperative values, mean thinning was 5.1 μm (*p* = 0.30, six weeks), 7.2 μm (*p* = 0.14, six months), 7.9 μm (*p* = 0.14, one year) and 6.3 μm (*p* = 0.21, latest follow-up) after A-CXL.

The longitudinal analysis of CCT measurements by Pentacam HR showed a significant thinning of the central cornea after A-CXL. After six weeks, a mean thinning of 12.6 μm (*p* = 0.006, LSD) was observed compared to preoperative values. Thereafter CCT did not change significantly over time, but remained significantly lower than the preoperative values with a mean thinning of 11.7 μm (*p* = 0.01, six months) and 10.1 μm (*p* = 0.039, one year). At the latest follow-up, the thinning of 9.1 μm was not significant (*p* = 0.053).

CCT measurements by Casia 2 (AS-OCT) showed lower postoperative measurements, without any significant thinning over time, with a similar evolution as TCT measurements. Compared to the preoperative values, there was a mean thinning of 3.4 μm (p = 0.48, six weeks), 4.8 μm (*p* = 0.32, six months), 5.3 μm (p = 0.32, one year) and 3.9 μm (*p* = 0.44, latest follow-up) after A-CXL.

**Fig. 3:**
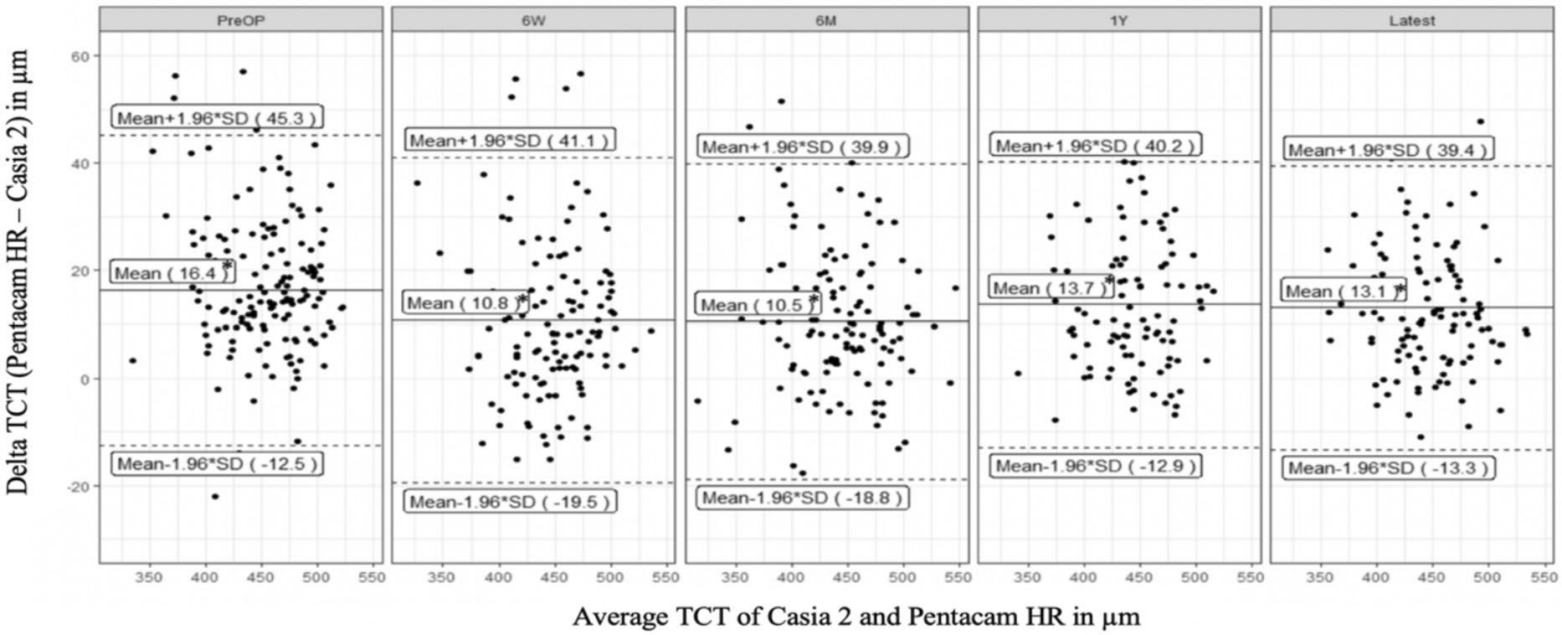
Bland–Altman-plots showing mean ± 1.96 SD of thinnest corneal thickness (TCT) ΔPentacam HR – Casia 2 in μm with significantly higher measurements by Pentacam HR pre-operatively (PreOP), at six weeks (6W), six months (6M), one year (1Y) and at the latest follow-up examination (Latest) after A-CXL. * indicating significance on a 0.05 level in paired samples t-test

**Fig. 4:**
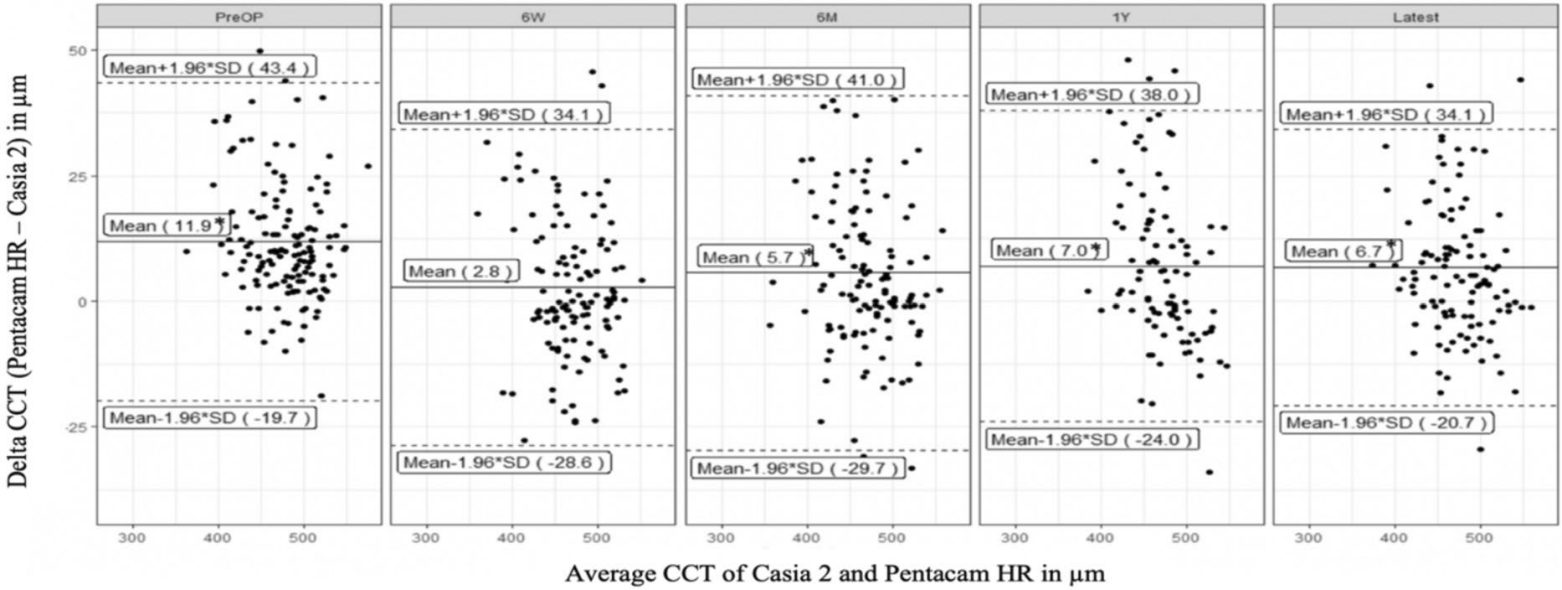
Bland–Altman-plots showing mean ± 1.96 SD of central corneal thickness (CCT) ΔPentacam HR – Casia 2 in μm with significantly higher measurements by Pentacam HR pre-operatively (PreOP), at six months (6M), one year (1Y) and at the latest follow-up examination (Latest) after A-CXL. There was no significant inter-device measurement difference six weeks (6W) after A-CXL. * indicating significance on a 0.05 level in paired samples t-test

Second, the measurement differences between Pentacam HR and Casia 2 AS-OCT were analyzed and the mean measurement differences in TCT and CCT between the two devices for each time point are presented in Table 2 and visualized in Fig. 3 (TCT) and Fig. 4 (CCT).

**Table 2:** Mean thinnest corneal thickness (TCT) and central corneal thickness (CCT) ± SD with numbers of eyes for both devices (Pentacam HR and Casia 2) and mean measurement difference (Pentacam HR–Casia 2) ± SD at different time points (before A-CXL (PreOP) and at six weeks (6W), six months (6 M), one year (1Y) and latest follow-up (Latest) after CXL) with two-sided p-values according to paired samples t-test

Date category	TCT Pentacam HR ± SD (μm)	TCT Casia 2 ± SD (μm)	Δ TCT (Pentacam HR – Casia 2) ± SD (μm)	CCT Pentacam HR ± SD (μm)	CCT Casia 2 ± SD (μm)	Δ CCT (Pentacam HR – Casia 2) ± SD (μm)	n
PreOp	460.8 ± 37.9	444.4 ± 39.6	16.4 ± 14.7* *p* < 0.001	483.8 ± 37.0	472.0 ± 39.2	11.9 ± 16.1* *p* < 0.001	147
6 W	450.1 ± 40.8	439.3 ± 40.7	10.8 ± 15.4* *p* < 0.001	471.2 ± 37.0	468.5 ± 38.9	2.8 ± 16.0 *p* = 0.054	127
6 M	448.0 ± 42.6	437.5 ± 44.0	10.5 ± 15.0* *p* < 0.001	472.2 ± 40.9	466.5 ± 42.7	5.7 ± 18.0* *p* < 0.001	126
1 Y	450.2 ± 37.5	436.5 ± 37.0	13.7 ± 13.5* *p* < 0.001	473.7 ± 33.4	466.7 ± 39.3	7.0 ± 15.8* *p* < 0.001	97
Latest	451.2 ± 38.7	438.1 ± 39.8	13.1 ± 13.4* *p* < 0.001	474.8 ± 37.6	468.0 ± 40.5	6.7 ± 14.0* *p* < 0.001	114

* indicating significance on a 0.05 level

There were significantly higher measurements of TCT by Pentacam HR at all time points compared to Casia 2 AS-OCT.

CCT measurements by Pentacam HR were significantly higher than the ones by Casia 2 at all time points (exception: six weeks after A-CXL), suggesting that there is a tendency for Pentacam HR to continuously measure higher CCT than Casia 2 AS-OCT. Strikingly, the best measurement agreement between both devices was found at six weeks after A-CXL with a mean difference of 2.8 µm (Fig. 4).

**Fig. 5:**
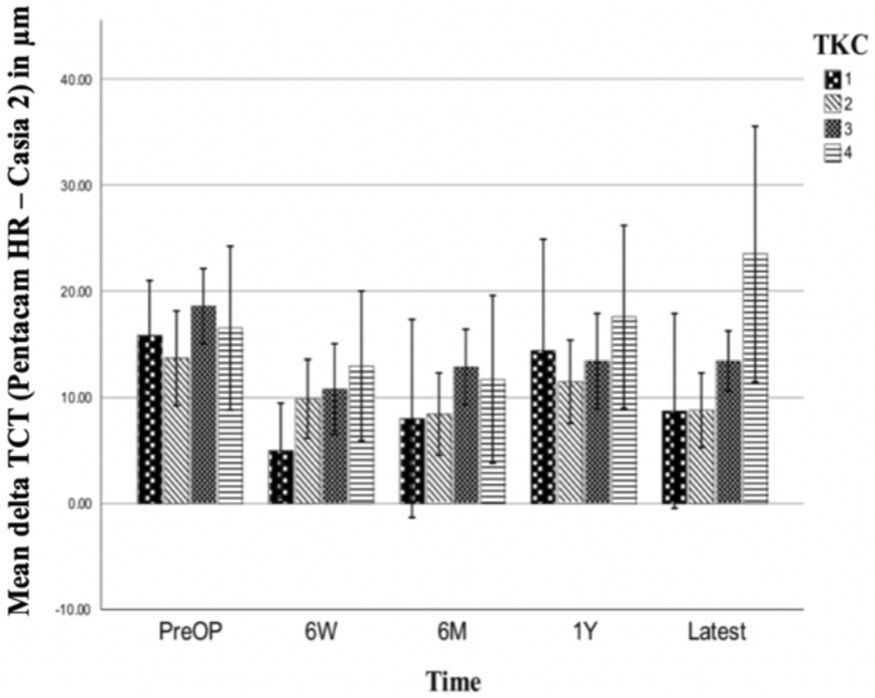
Mean thinnest corneal thickness (TCT) measurement deviations of Pentacam HR–Casia 2 split into topographic keratoconus classification (TKC) stages 1–4, given with error bars indicating the 95% confidence interval

**Fig. 6:**
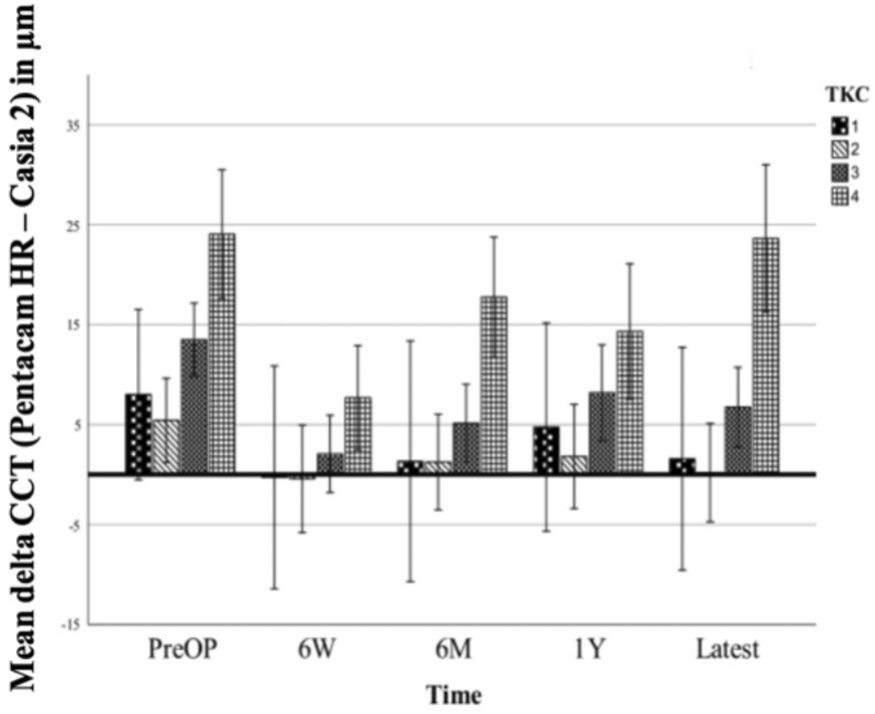
Mean CCT measurement deviations of Pentacam HR–Casia 2 split into TKC stages 1–4, given with error bars indicating the 95% confidence interval

The third analysis dealt with measurement differences between the two devices divided into the different TKC stages provided by Pentacam software. Whenever eyes were classified in an interim stage, the stage was rounded up, e.g. an eye classified as TKC 1.5 was considered as TKC 2. The results are presented in Fig. 5 for TCT and Fig. 6 for CCT. The corresponding absolute numbers are shown in Table 3.

**Table 3:** Thinnest corneal thickness (TCT) and central corneal thickness (CCT) measurement differences (mean ± SD) of Pentacam HR–Casia 2 in μm split into topographic keratoconus classification (TKC) stages 1 to 4, provided by Pentacam HR software, pre-operatively (PreOP), at six weeks (6W), six months (6M), one year (1Y) and at the latest follow-up examination (Latest) after A-CXL

Date	TKC	Δ TCT (Pentacam HR–Casia 2) Mean ± SD in μm	n	Δ CCT (Pentacam HR–Casia 2) Mean ± SD in μm	n
PreOP	1	15.8 ± 8.1	12	8.0 ± 6.1	12
	2	13.7 ± 15.5	49	5.4 ± 13.8	49
	3	18.6 ± 14.3	65	13.5 ± 12.6	65
	4	16.5 ± 16.9	21	24.0 ± 25.2	21
6W	1	5.0 ± 4.8	7	-0.3 ± 3.0	7
	2	9.9 ± 9.9	30	-0.4 ± 8.8	30
	3	10.8 ± 16.1	58	2.1 ± 14.7	58
	4	13.0 ± 19.5	32	7.7 ± 23.1	32
6 M	1	8.0 ± 6.8	7	1.3 ± 7.0	7
	2	8.4 ± 11.8	38	1.3 ± 11.8	38
	3	12.9 ± 13.3	56	5.1 ± 16.0	56
	4	11.7 ± 11.3	25	17.8 ± 22.8	25
1Y	1	14.4 ± 12.6	8	4.8 ± 12.2	8
	2	11.5 ± 10.8	32	1.8 ± 10.0	32
	3	13.4 ± 16.9	38	8.2 ± 15.8	38
	4	17.6 ± 17.9	19	14.3 ± 21.9	19
Latest	1	8.7 ± 9.9	7	1.6 ± 6.9	7
	2	8.8 ± 10.4	36	0.2 ± 10.0	36
	3	13.4 ± 10.4	55	6.7 ± 12.7	55
	4	23.5 ± 22.6	16	23.6 ± 14.9	16

The mean TCT measurement difference between Pentacam HR—Casia 2 increased with increasing KC stage and accounted for 10.4 μm | 10.5 μm | 13.8 μm | 16.5 μm for TKC 1|2|3|4, respectively. The difference was significant for TKC 4 versus TKC 2 (*p* = 0.04, ANOVA).

A mean CCT measurement difference between Pentacam HR–Casia 2 of 3.1 μm | 1.7 μm | 7.1 μm | 17.5 μm for TKC 1|2|3|4 could be observed. Over time there were significantly higher (*p* < 0.001, ANOVA) measurement differences of 14.4 μm | 15.8 μm | 10.4 μm in TKC 4 eyes compared to TKC 1|2|3 eyes. The difference of 5.5 μm in TKC 3 eyes compared to TKC 2 eyes (*p* = 0.001) was also significant. There were no other significant differences in measurement differences for other TKC stages.

**Fig. 7:**
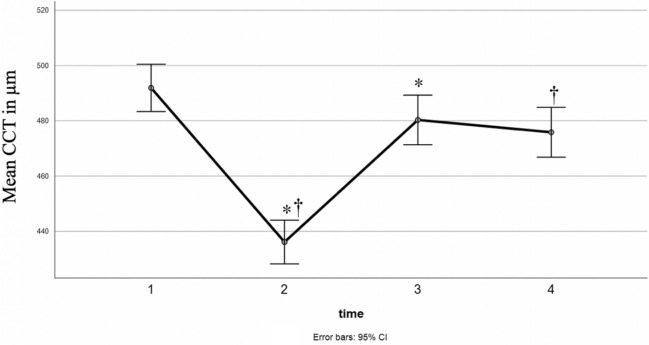
Mean with 95% confidence interval (CI) error bars of central corneal thickness (CCT) (given in μm, y-axis) measured intraoperatively using ultrasound (Pachymeter SP-3000) before epithelial abrasion (1), after epithelial abrasion (2), after Riboflavin application (3) and after ultraviolet A irradiation without epithelium (4). A significant decrease of CCT after epithelial abrasion was followed by a significant increase after Riboflavin saturation. CCT directly after A-CXL was measured significantly lower than before. * indicates significant (*p* < 0.05, ANOVA) changes in CCT compared to the measurement before, † indicates significant (*p* < 0.05) changes in CCT compared to (1)

Intraoperative CCT evolution was provided by ultrasound pachymetry measurements to ensure that CCT did not fall below 400 μm at any time and to provide continuous measurements even intraoperatively to supplement the pre- and postoperative findings with another measurement modality. The longitudinal evolution and absolute CCT values are presented in Fig. 7.

The mean CCT decreased significantly from 492 to 436 µm after epithelial abrasion (*p* < 0.001, ANOVA) and increased significantly to 480 µm (*p* < 0.001) after riboflavin application. Finally, CCT was measured at 476 µm after A-CXL and, thus, significantly thinner than preoperatively (*p* < 0.001), so that there was a mean decrease of 16 μm in CCT during the whole A-CXL process as measured by ultrasound pachymetry.

Few patients showed a post-A-CXL haze formation of stages 0, 0.5 and 1 according to the corneal haze stages of Fantes et al. [23]

**Fig. 8:**
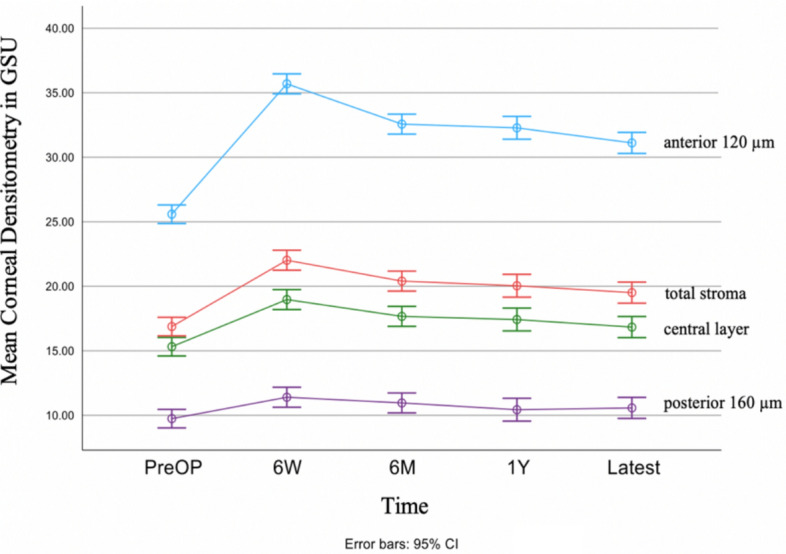
Mean central 0.0 to 2–0 mm corneal densitometry in grayscale units (GSU) is shown over time (PreOP, 6W, 6 M, 1Y, Latest) with error bars indicating the 95% confidence interval (CI) interval, separately for the anterior 120 μm of the cornea, the central layer, the posterior 160 μm and for the total depth of the cornea

The longitudinal CD evolution, as assessed by Pentacam HR, is shown in Fig. 8. The correlation between CD and CCT was also analyzed and the results are shown in Table 4.

**Table 4:** Pearson correlation between central corneal thickness (CCT) by Pentacam and central 0.0–2.0 mm corneal densitometry (CD) split into anterior 120 μm, central layer, posterior 60 μm and total corneal thickness

Date	CCT Pentacam HR		Dens Avg, Ant. 120 µm, Annulus 0.0–2.0 mm	Dens Avg, Center, Annulus 0.0–2.0 mm	Dens Avg, Post. 60 µm, Annulus 0.0–2.0 mm	Dens Avg, Tot.Th., Annulus 0.0–2.0 mm
PreOP	CCT Pentacam HR	Pearson Correlation Sig. (2-tailed) N	−.045 .588 147	−.156 .060 147	.095 .251 147	−.040 .627 147
6W	CCT Pentacam HR	Pearson Correlation Sig. (2-tailed) N	.133 .137 127	−.048 .595 127	.005 .956 127	.078 .381 127
6 M	CCT Pentacam HR	Pearson Correlation Sig. (2-tailed) N	−.235^**^ .008 126	−.357^**^ < .001 126	−.263^**^ .003 126	−.295^**^ < .001 126
1Y	CCT Pentacam HR	Pearson Correlation Sig. (2-tailed) N	−.069 .499 97	−.136 .183 97	.226^*^ .026 97	−.052 .613 97
Latest	CCT Pentacam HR	Pearson Correlation Sig. (2-tailed) N	−.150 .110 114	−.233^*^ .013 114	.114 .225 114	−.151 .109 114

* indicating significance on a 0.05 level

The CD in the central 0.0 to 2.0 mm annulus increased significantly to a maximum six weeks after A-CXL and decreased afterwards without reaching pre-A-CXL levels in all layers of the cornea as defined by Pentacam HR. The greatest changes in CD could be observed in the anterior 120 μm of the cornea. Starting from a mean CD of 25.6 ± 3.2 GSU before A-CXL in the anterior 120 μm layer (blue), the CD increased significantly by 10.1 GSU (*p* < 0.001, ANOVA) to a maximum of 35.7 ± 8.3 GSU six weeks after A-CXL. CD values decreased by 3.1 GSU (*p* = 0.006) to 32.6 ± 8.6 GSU six months after A-CXL. Thereafter, there were no significant changes in CD, resulting in a mean CD value of 31.1 ± 7.2 GSU at the latest follow-up, which was 5.5 GSU higher than before A-CXL (*p* < 0.001).

CD values of the central (green) and posterior (purple) layer followed a similar pattern with peaking values at six weeks after A-CXL and decreasing values thereafter, which remained significantly higher than preoperatively at latest follow-up (*p* < 0.001 central layer, *p* = 0.016 posterior layer, Fig. 8).

There was no high correlation between CCT and central CD at any time point, suggesting that haze formation is not correlated with corneal thickness measurements (Table 4).

## Discussion

The main question to be answered with this study was whether the corneal thinning after A-CXL observed in Scheimpflug-based imaging was also present in AS-OCT thickness measurements or whether it was rather due to measurement artifacts possibly occurring in Scheimpflug imaging because of postoperative haze formation. There is no clear global definition of ectasia progression. However, in addition to other tomographic parameters like surface curvature, decreasing corneal thickness may be used as an indicator of KC progression [24]. Previous studies even suggested decreasing pachymetry in combination with the posterior radius as the most sensible markers for KC progression, indicating progression even when Kmax has not increased yet. They concluded, that Kmax as a single parameter is not reliable when evaluating KC Progression, but a combination of parameters as used for the ABCD staging and especially a comparison of serial tomographic assessments are crucial to detect progression reliably [24, 25]. Therefore, reliable measurements are crucial to properly evaluate corneal thickness changes. Previous studies have shown that corneal thickness measurements obtained by Scheimpflug-based imaging of the cornea are more susceptible to measurement inaccuracies than other methods such as AS-OCT [16–18]. Thus, corneal thickness measurements obtained by different measurement techniques should not be used interchangeably. The current study found a significant corneal thinning up to one year after A-CXL for CCT and TCT measurements by Pentacam HR, as described in previous studies [7, 15]. However, this trend, which might be considered a “pseudoprogression”, was not reproducible with statistical significance for AS-OCT Casia 2 measurements of CCT and TCT. The relative reduction of TCT and CCT was higher in the Pentacam HR compared to the Casia 2 measurements at all times, supporting the observation that the Pentacam HR measures significantly lower TCT and CCT after A-CXL. Antonios et al. [26] have found similar results after standard CXL, suggesting that Scheimpflug-based measurements overestimate corneal thinning after CXL.

Baiao et al. [27] also compared the pachymetry measurements of Scheimpflug-based imaging and spectral-domain AS-OCT (MS-39, CSO, Italy) and found significantly lower CCT measurements up to 3 months after customized CXL by Pentacam HR while the AS-OCT did not measure significantly lower CCT measurements. The current study found similar results in CCT measurements after A-CXL with (1) significantly lower CCT measured by Pentacam HR and (2) no significantly lower CCT measured by AS-OCT Casia 2. However, the studies differed in the findings that Pentacam HR measurements of CCT and TCT were higher than Casia 2 measurements in the current study, whereas Mendes Baiao et al. found Pentacam HR CCT and TCT measurements even lower than those of the MS-39 AS-OCT [27]. These differences between the two studies are likely to be attributable to the different CXL protocols applied and the different kinds of OCT devices that were used. Wang et al. [28] recently reported, that TCT and CCT measurements using a spectral-domain OCT were higher than those obtained by a swept-source OCT. The postoperative differences between the two measurement modalities themselves may also be attributed to an increase in the corneal index of refraction after CXL (data presented by Prof. Dr. C. Roberts, 2nd World Keratoconus Congress, Athens 2025).

The TKC stage-dependent analysis showed a trend towards higher measurement deviations between Pentacam HR and Casia 2 in eyes of higher TKC stages, particularly for CCT measurements. This supports the observations of reduced reliability, especially of the Scheimpflug-based imaging in advanced TKC stages [17]. However, for TCT, this study could only find minor effects of the TKC stage on the measurement deviation between Pentacam HR and Casia 2. There were significantly lower CCT and TCT measurements at all time points when comparing Casia 2 AS-OCT and Pentacam HR (exception: CCT at six weeks after A-CXL). Six weeks after A-CXL we did not find significantly lower measurements in CCT with the Casia 2, as the relative reduction in CCT by Casia 2 was smaller (−0.7%) than the one in CCT measurements by Pentacam HR when comparing CCT before and six weeks after the surgery (−2.6%). This resulted in aligning CCT measurements six weeks after A-CXL without a significant delta between both devices. Corneal densitometry peaked at this time and might contribute to the high difference in relative CCT change measured by both devices [26]. While Antonios et al. [26] did not find significant differences in pachymetric measurements using OCT and Scheimpflug-based imaging before CXL, Kanellopoulos et al. [19] found highest CCT measurements using US, followed by Scheimpflug imaging and lowest values using OCT, suggesting that even before surgery the pachymetric measurements cannot be used interchangeably. The fact that Scheimpflug-based imaging devices provide higher values for corneal thickness has been observed in previous studies as well [18, 19]. This might be explained by the different measurement modalities used by Casia 2 and Pentacam HR to acquire their measurements. Pentacam HR uses blue light at a wavelength of 475 nm which is more susceptible to scattering than the infrared light with a longer wavelength of 1310 nm used by the Casia 2 AS-OCT, particularly at optical interfaces like the tear film and the posterior corneal surface. For this reason, the location of the anterior and posterior corneal surface might be more prone to measurement fluctuations in the Pentacam HR than in Casia 2, thus contributing to slightly higher thickness measurements by the Pentacam HR [17, 19, 29]. Herber et al. [30] have compared the reliability of different measuring techniques and observed lower corneal thickness measurements by OCT. They concluded, that SS-OCT and Scheimpflug-based measurements cannot be used interchangeably and SS-OCT measurements seem to be more reliable and should therefore be preferred in assessing corneal thickness in KC eyes. However, they also pointed out, that there is no consensus on whether there are conversion factors applicable and measurement deviations might be dependent on various factors such as age.

In order to compare corneal thickness measurements over time, they should be measured by the same device, preferably by OCT, as this seems to be less susceptible to measuring artefacts.

However, as both measuring techniques are reliable and offer advantages and disadvantages, they should be combined to evaluate KC progression. Decreasing CCT and TCT measurements should not be interpreted as stand-alone parameters, as this could lead to premature conclusions of KC progression.

This study has shown that central corneal thickness increased after a 20-min saturation period with 0.1% riboflavin hydroxypropyl-methylcellulose (HPMC) eye drops. There was a thickness increase of 10.1% during the saturation phase, which is consistent with an increase between 5 and 26% reported in previous studies [31]. A significant thinning of the cornea after riboflavin application as described in other studies [32, 33] could not be observed, which might be explained by the improved riboflavin formulation with HPMC instead of dextran. The HPMC has previously shown to form a thicker precorneal biofilm, resulting in less evaporation and preventing corneal thinning [31]. The removal of the eyelid speculum, allowing the patient to blink, also contributes to less dehydration of the cornea.

This is a favorable effect since one limitation of A-CXL is the corneal thickness which should not fall below 400 μm [34]. Immediately after A-CXL, CCT had decreased significantly compared to preoperative CCT values, however, the finally measured reduction in CCT (−16 µm) compared to preoperative measurements was less than the decrease in thickness that was initially observed after the abrasion of the corneal epithelium (−56 µm).

Previous studies have shown a comparable efficacy and safety of A-CXL and the standard CXL protocol [35], with A-CXL providing higher patient comfort by shortening the duration of the treatment and therefore also optimizing clinical workflow. Mazzotta et al. [36] have shown that it is still a safe and effective treatment up to 5 years after A-CXL, suggesting that this protocol could emerge to a new standard protocol.

The CD increased significantly, reaching its maximum six weeks after A-CXL in this study, which was also reported in other studies [37, 38]. Afterwards CD decreased significantly, however we observed increased CD values up to more than one year after A-CXL. Increased CD has been associated with corneal haze, an opacification of the anterior corneal stroma, often observed after corneal surface interventions such as A-CXL. After A-CXL, increased CD and haze may be the result of complex corneal wound healing processes, including inflammatory responses, leading to myofibroblast activation and extracellular matrix production, resulting in a higher amount of light scattering [39].

In contrast to the current study, Prinz et al. [40] found an increase in CD only after conventional but not after A-CXL.

Turhan et al. [41] compared the longitudinal evolution of corneal haze after CXL protocols with different irradiation intensities and concluded that higher radiation intensity may lead to greater haze formation in the early postoperative phase with a faster regression afterwards.

Badawi [39] found a later regression of corneal haze after A-CXL compared to the standard CXL protocol which is consistent with the increased CD values seen in our study population even more than one year after A-CXL.

Peskina et al. [42] described that densitometry values in the anterior central stroma only returned to preoperative levels 24 months after A-CXL, after reaching a maximum six weeks after A-CXL. The densitometry values remained elevated for a long time, particularly in the stromal areas that had previously shown the highest CD-increase, while CD of the posterior areas returned to baseline levels after only three to six months. Fard et al. [38] found deep-stromal CD changes in the A-CXL group and attributed this observation to the higher irradiation intensity compared to the conventional protocol, leading to a deeper penetration and thus a deeper CD-increase.

In the current study, the effects of A-CXL on CD were most pronounced in the anterior 120 μm layer, as might be expected since A-CXL has its greatest effect in the anterior 200 μm [43].

In contrast to Antonios et al. [26], the current study did not find any correlation between CD and CCT measured by Pentacam HR. It cannot be assumed that an increased CD value with a higher amount of light scattering affects the CCT measurements of Pentacam HR, resulting in either significantly higher or lower CCT values.

Finally, the possible corneal thinning after A-CXL within the first postoperative year as measured by Pentacam HR, was not reproducible with the Casia 2 AS-OCT. Although the latter showed a tendency to lower postoperative corneal thickness measurements indicating a corneal thinning, this observation did not reach statistical significance. This led to the conclusion that according to our results and a higher reliability of the OCT measurements in KC eyes [30], Pentacam HR seems to be more likely to overestimate a possible post-A-CXL corneal thinning.

Therefore, especially Scheimpflug-based pachymetry measurements cannot be interpreted as indicative of the long-term stabilization of KC within the first year after A-CXL. They should always be interpreted with care and never be used as the main parameter to evaluate the success or failure of A-CXL treatment.

The higher number of male patients included in this study, creating a gender imbalance, might be a limitation of this study. However, KC is more often diagnosed in male patients, and therefore the higher number of male patients reflects the clinical situation. Further studies on gender differences in A-CXL outcome should be performed in the future.

Especially in the TKC stage dependent analysis, the number of patients in each stage was not very high, further studies with more eyes included for each stage would be interesting for stage-dependent analysis.

Another limitation of the current study was the precise location of the central cornea during the intraoperative ultrasound pachymetry measurements showing significantly lower CCT values post- than preoperatively. As it was estimated manually by the examiner, this may have led to slight variations between the measurements.

## Data Availability

No datasets were generated or analysed during the current study.
